# Effectiveness of educational video on deaf people’s knowledge and skills for cardiopulmonary resuscitation: a randomized controlled trial^*^

**DOI:** 10.1590/1980-220X-REEUSP-2022-0227en

**Published:** 2023-05-22

**Authors:** Nelson Miguel Galindo, Guilherme Guarino de Moura Sá, Lívia Moreira Barros, Magda Milleyde de Sousa Lima, Sônia Maria Josino dos Santos, Joselany Áfio Caetano

**Affiliations:** 1 Instituto Federal de Educação, Ciência e Tecnologia de Pernambuco, Departamento de Enfermagem, Pesqueira, PE, Brazil.; 2 Instituto Federal de Educação, Ciência e Tecnologia de Pernambuco, Departamento de Enfermagem, Belo Jardim, PE, Brazil.; 3 Universidade da Integração Internacional da Lusofonia Afro-Brasileira, Instituto de Ciências da Saúde, Redenção, CE, Brasil.; 4 Universidade Federal do Ceará, Departamento de Enfermagem, Fortaleza, CE, Brasil.; 5 Universidade da Paraíba, Departamento de Enfermagem, João Pessoa, PB, Brasil.

**Keywords:** Persons with Hearing Impairments, Sign Language, Cardiopulmonary Resuscitation, Knowledge, Instructional Film and Video, Health Education, Personas con Deficiencia Auditiva, Lengua de Signos, Reanimación Cardiopulmonar, Conocimiento, Película y Video Educativos, Educación en Salud, Pessoas com Deficiência Auditiva, Língua de Sinais, Reanimação Cardiopulmonar, Conhecimento, Filmes e Vídeos Educativos, Educação em Saúde

## Abstract

**Objective:**

To analyze the effectiveness of an educational video on deaf people’s knowledge and skills about cardiopulmonary resuscitation.

**Method:**

A randomized trial, carried out at three schools with 113 deaf people (control group = 57 and intervention group = 56). After the pre-test, the control group was exposed to the lecture, while the intervention group was exposed to the video. The post-test occurred immediately after the intervention and was repeated after 15 days. A validated instrument was used, with 11 questions, presented in video/Libras (to enable understanding by deaf people) and in written/printed form (to record the answers).

**Results:**

In the analysis of knowledge, the median of correct answers in the pre-test was similar between the groups (p = 0.635), while the intervention group had a higher accuracy in the immediate post-test (p = 0.035) and after 15 days (p = 0.026). In the skill analysis, the median of correct answers in the pre-test was higher in the control group (p = 0.031). There was no difference in the immediate post-test (p = 0.770), and there was a higher accuracy in the intervention group in the post-test after 15 days (p = 0.014).

**Conclusion:**

The video proved to be effective in increasing deaf people’s knowledge and skills about cardiopulmonary resuscitation. Brazilian Registry of Clinical Trials: RBR-5npmgj.

## RESUMO

**Objetivo:**

Analisar a efetividade de vídeo educativo no conhecimento e habilidade de surdos acerca da ressuscitação cardiopulmonar.

**Método:**

Ensaio randomizado, realizado em três escolas com 113 surdos (grupo controle = 57 e grupo intervenção = 56). Após o pré-teste, o grupo controle foi exposto à aula expositiva, enquanto o grupo intervenção foi exposto ao vídeo. O pós-teste ocorreu imediatamente depois da intervenção, e foi repetido após 15 dias. Foi utilizado instrumento validado, com 11 questões apresentadas em vídeo/libras (para viabilizar compreensão dos surdos) e de forma escrita/impressa (para registro das respostas).

**Resultados:**

Na análise do conhecimento, a mediana dos acertos do pré-teste foi semelhante entre os grupos (p = 0,635), enquanto houve maior acerto do grupo intervenção no pós-teste imediato (p = 0,035) e após 15 dias (p = 0,026). Na análise da habilidade, a mediana dos acertos do pré-teste foi maior no grupo controle (p = 0,031). Não houve diferença no pós-teste imediato (p = 0,770), e houve maior acerto do grupo intervenção no pós-teste após 15 dias (p = 0,014).

**Conclusão:**

O vídeo mostrou-se efetivo no aumento do conhecimento e habilidade de surdos acerca da ressuscitação cardiopulmonar. Registro Brasileiro de Ensaios Clínicos: RBR-5npmgj.

## RESUMEN

**Objetivo:**

Analizar la efectividad de un video educativo sobre el conocimiento y la habilidad de las personas sordas acerca de la reanimación cardiopulmonar.

**Método:**

Ensayo aleatorizado, realizado en tres escuelas con 113 personas sordas (grupo control = 57 y grupo intervención = 56). Después de la prueba previa, el grupo de control fue expuesto a la conferencia, mientras que el grupo de intervención fue expuesto al video. La posprueba se realizó inmediatamente después de la intervención, y se repitió a los 15 días. Se utilizó un instrumento validado, con 11 preguntas presentadas en video/Libras (para que los sordos comprendan) y en forma escrita/impresa (para registrar las respuestas).

**Resultados:**

En el análisis de conocimientos, la mediana de aciertos en la preprueba fue similar entre los grupos (p = 0,635), mientras que el grupo intervención tuvo mayor acierto en la posprueba inmediato (p = 0,035) y después 15 días (p = 0,026). En el análisis de habilidades, la mediana de aciertos en la preprueba fue mayor en el grupo control (p = 0,031). No hubo diferencia en la posprueba inmediato (p = 0,770), y el grupo intervención tuvo más éxito en la posprueba a los 15 días (p = 0,014).

**Conclusión:**

El video demostró ser efectivo para aumentar el conocimiento y las habilidades de las personas sordas sobre la reanimación cardiopulmonar. Registro Brasileño de Ensayos Clínicos: RBR-5npmgj.

## INTRODUCTION

According to the American Hearth Association (AHA), there is importance in teaching laypersons regarding cardiopulmonary resuscitation (CPR)^(1)^, since the assistance provided by people without training in the health area is associated with increased survival of victims of cardiorespiratory arrest (CRA) in the pre-hospital setting^(2)^.

When considering that technological resources are used for disseminating health information, the relevance of researches that investigate the effectiveness of existing technologies, which include teaching CPR for laypeople, stands out^(3)^. In this way, once such research is carried out, the choice and use of technological options can be supported by scientific evidence.

Among the existing technologies, the video entitled “Cardiorespiratory arrest: how to act to save” stands out, which was built in animation format and consists of an inclusive technology, as it has audio narration and in the Brazilian Sign Language (Libras)^(3)^. The presence of Libras makes the video compatible with the understanding of an audience that has the cognitive and motor capacity to act in the face of a CRA, but faces a barrier to accessing health information: deaf people.

Deaf people, who have hearing loss greater than 40dB, account for more than 430 million people worldwide and, according to the World Health Organization, will reach 700 million by 2050^(4)^. In view of their high number, deaf people are included in all sectors of society, so they can witness a CRA that occurs outside the hospital and, therefore, need to know how to act to save. However, the chance of knowing the correct conduct to be adopted in the face of a CRA is low, since deaf people do not experience inclusion in health services nor are they the target audience of health education actions, as observed in a literature review which analyzed studies on the main difficulties faced by deaf people in accessing health services^(5)^.

Thus, researches that contemplate CPR teaching and the investigation regarding the effectiveness of educational technologies aimed at deaf people are pertinent to contribute to reducing mortality in CRA. Moreover, since health education is present in nursing work practice^(6)^, it is noteworthy that research that contemplates the verification of the effectiveness of educational technology for deaf people about CPR is relevant for these health professionals, who they will be able to use it in their educational interventions and in the multiplication of information about CPR for deaf people.

According to an integrative review, existing technologies used by health professionals with the deaf public are mainly about cancer, self-management of hearing loss, sexual and reproductive health education, high blood pressure, accessibility of sites with health information. Regarding the CPR theme, the authors identified only the video “Cardiorespiratory arrest: how to act to save”^(7)^. In addition to this, another integrative review, which investigated studies on technologies for health education for death people, did not find research that tested the use of an educational video in Libras to teach death people about CPR^(8)^, which confirms the existing knowledge gap.

Furthermore, there are still gaps in the literature about the effectiveness of educational technologies aimed at death people, since existing studies contemplate other profiles, mainly laypeople without hearing disorders^(9,10,11)^.

Given the above, the present study aimed to analyze the effectiveness of an educational video in deaf people’s knowledge and skills about CPR.

## METHODS

### Study Design

This is a randomized, controlled, single-blind clinical trial, carried out with two groups allocated at a 1:1 ratio. The study was reported based on the Consolidated of Reporting Trials (CONSORT) for Randomized Trials of Nonpharmacologic Treatments^(12)^. The study has the Brazilian Clinical Trials Registry with primary identifier: RBR-5npmgj.

### Population

The population consisted of 676 deaf students belonging to three educational institutions located in the city of Fortaleza, CE, Brazil.

### Place

Data were collected at three public schools, located in the city of Fortaleza, Ceará, Brazil. The first was the only state school that offered elementary and high school exclusively for deaf people. The second also belonged to the state network of Ceará and did not have exclusive education for death people, but allocated specific vacancies for deaf people in high school. And the third consisted of a Catholic philanthropic institution with exclusive vacancies for deaf people, in kindergarten, and elementary, high and vocational schools.

### Selection Criteria

Students enrolled in elementary or high school at one of the institutions where data collection took place and not having a limitation that would make participation in the lecture or use of the video unfeasible were included. Students missing school activities during the data collection period, who did not answer all the questions on the data collection instrument, or did not have physical fitness, due to disability or movement limitation, to participate in CPR practice, were excluded.

### Sample Definition

The sample calculation was based on the formula for studies comparing groups^(13)^, in which the confidence coefficient adopted was 95%; test power was 80%; the prevalence of the outcome in the control group was 50%; and the prevalence of the outcome in the intervention group was 75%. From these values, a sample size of 55 individuals was obtained for each group (control and intervention), which totaled a minimum of 110 participants to integrate the study sample.

**Figure 1.**
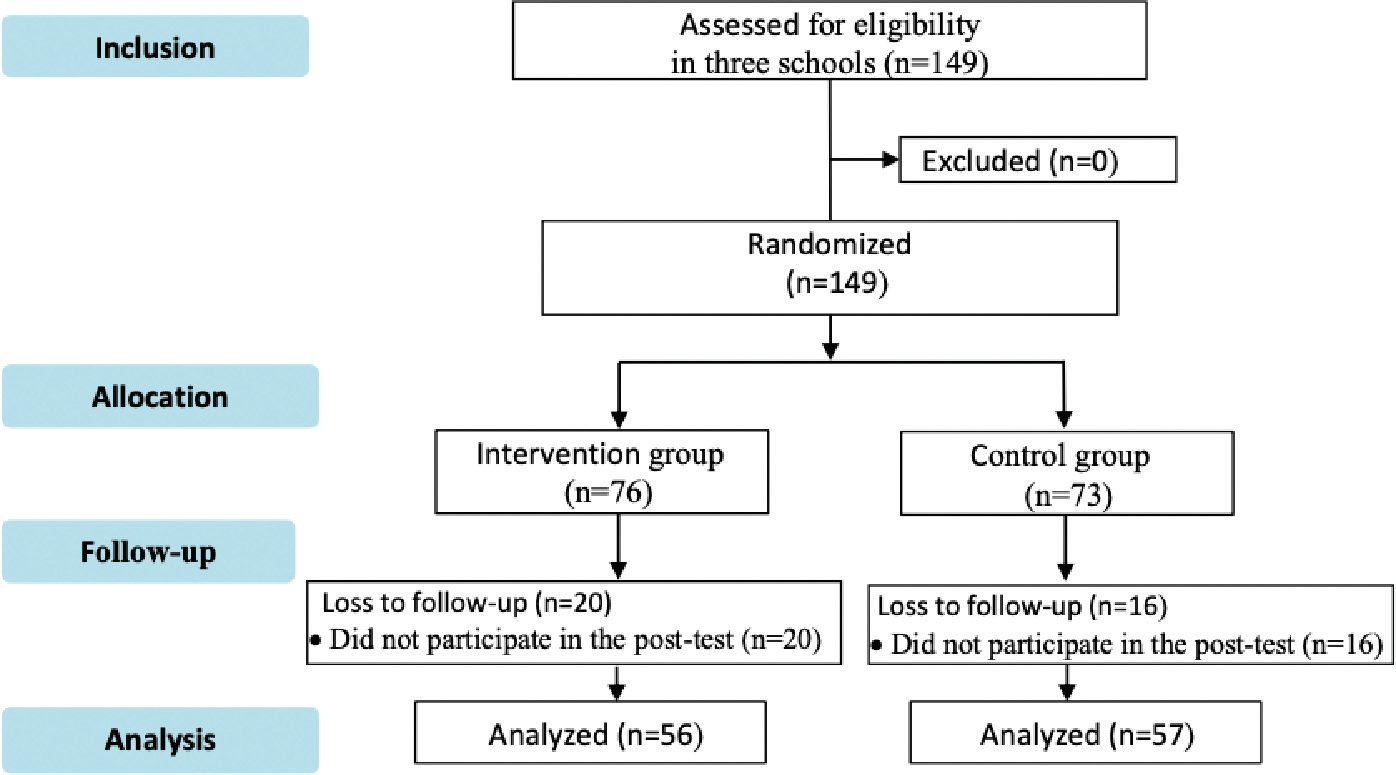
Participant selection flowchart. Fortaleza, CE, Brazil, 2018.

When considering the possibility of losses, during the operationalization of data collection, 149 participants were recruited (73 from the control group and 76 from the intervention group), of which 36 were discontinued from the study (16 from the control group and 20 from the intervention group) for not attending the post-test performed 15 days after the intervention. Thus, the final sample consisted of 113 participants: 57 in the control group and 56 in the intervention group (Figure 1).

### Data Collection

Data collection was carried out from October to December 2017 so that it lasted one month in each of the three schools that were the study settings. To minimize possible contamination of the control group, which was exposed to the lecture, with information from the video, which consisted of an intervention applied only to the intervention group, collection took place at each school, starting chronologically with the control group participants. Only upon conclusion of the post-test with the control group, collection with the intervention group took place at each school so that even if there was sharing of information about the video, there would be no interference in the control group results, since collection with this one had already been closed. Study participants were selected by the educational institutions themselves, based on the availability of opportune times in the classes, according to the class schedules. Thus, participants were divided into 12 subgroups, according to educational institutions’ availability to release students from classes for data collection feasibility. It should be noted that the number of students that made up each subgroup was determined by participation availability so that the subgroups were composed of numbers ranging from nine to 14 participants.

Cluster randomization was used, in which each cluster was one of the 12 subgroups of students, and the R software was used to, with a 1:1 allocation rate, define the six groups of deaf people that would be included in the control group (CG) and the six that would belong to the intervention group (IG). To this end, a list of 12 subgroups was used, ordered in chronological sequence of collection schedule, and the numerical sequence obtained in R randomly determined which were allocated to the CG and IG.

The study took place in rooms reserved by the schools’ coordinators. The structural organization of the rooms was standardized, with chairs in a semicircle so that all participants were exposed to similar scenarios. It should be noted that the 10 participants, five from the IG and five from the CG, who had a hearing aid, but did not use it when participating in the research, not because there was a prohibition or restriction on using said device, but because death people spontaneously removed it when entering the school environment on a daily basis, as it was a place with routine communication in Libras, in which using hearing aids was considered unnecessary by death people themselves.

Upon arriving in the room, death students were invited to sit down and the researcher explained about the study, with translation into Libras performed by an interpreter. Then, participants signed the Informed Consent Form (ICF) and an attendance list, which included a numerical order for each signature so that the sequence of signed names assigned each participant a number. It should be noted that, during the operationalization of the study, the numbers were not repeated, thus each participant had a unique numerical code, determined by the sequence of signatures.

Then, participants answered a sociodemographic and clinical assessment instrument with the variables: age, marital status, existence of children, previous participation in class or previous contact with some educational material on the subject, previous experience with CPR, use of hearing aids and degree of right and left hearing loss. It should be noted that the referred degree of hearing loss was triangulated with information present in existing medical report and made available by the school secretariats.

Then, theoretical and practical pre-tests were carried out. Outcomes surveyed were CPR knowledge and skill. Thus, pre- and post-test measurements were performed using previously validated instruments. The instrument for verifying knowledge was built and validated in Libras, consisting of 11 questions about knowledge in CPR, such as CRP identification, CRP severity, right time to call for help, SAMU number, right time to perform compressions chest, victim positioning: hard and flat site, chest site for compression, position of rescuer for chest compression, strength to perform chest compression, rotation of who performs CPR and when to stop CPR. Next to each question there were five answer options (multiple choice), with only one correct alternative^(14)^.

In this regard, after the projection of each question and alternatives, the instrument designed in Libras was paused so that everyone could simultaneously record their answer on the printed part of the instrument.

After completing the theoretical pre-test, a skill pre-test took place. This stage took place in the same environment as the theoretical pre-test with the presence of only the participant, the researcher and the interpreter. Practice was carried out in a Prestan® adult CPR simulator. After being called individually to practice, each participant was instructed to freely perform in the simulator what they would do to help someone whose heart had stopped. It is noteworthy that skills assessments were recorded with a smartphone.

Upon completing the pre-tests, participants in the IG were submitted to video use on CPR. The video was built based on the Gagne’s Nine Events of Instruction theoretical framework, with a duration of 7 minutes and 30 seconds, with information regarding CRA severity and identification, the correct way to ask for help and how to perform high-quality CPR. Its content was based on the guidelines of the Brazilian Society of Cardiology of 2013^(15)^ and the AHA of 2015^(16)^, and was validated in a previous study, carried out with nurses and deaf students^(3)^. When applying the intervention, it was initially explained to participants that the video would show the correct way to help someone whose heart stopped, and attention and concentration were requested to watch the video. Subsequently, projection occurred only once, without re-presenting excerpts. No space was opened for participants to clarify doubts, since the effectiveness of teaching subsidized by the video was under assessment.

CG participants were exposed to content explanation about CPR in an expository class with practical demonstration. The content taught included CRA severity, the correct way to identify the condition, call for help and perform CPR, according to the protocol for laypersons of the Brazilian Society of Cardiology of 2013 and the AHA of 2015^(15,16)^. Content was taught by a nurse with experience in emergency care, with teaching experience on the subject, and occurred in an expositive way, for approximately 10 minutes, with translation into Libras performed by an interpreter. The resource used was the same simulator used in the practical pre-test to demonstrate CPR.

After the video intervention, applied to the IG, and the lecture with practical demonstration of CPR, applied to the CG, participants performed theoretical and practical post-tests. These occurred similarly to pre-tests and were carried out immediately after carrying out the educational strategy and repeated 15 days later. Outcome measurement in a 15-day interval is justified based on a scientific finding that compared teaching strategies and analyzed the effectiveness of learning, longitudinally, in three moments (immediately after, 15 and 30 days). The results of that study showed that, although the experimental group had better results, decline over time (15 and 30 days) was similar for both groups^(17)^.

Blinding occurred with the evaluators of pre- and post- practical tests. To this end skills assessment footage records were analyzed by two PhD teachers with experience in care and teaching in Emergency and Intensive Care, respectively. The two evaluators watched each footage together, projected on a 32-inch television, in a private environment, and filled out a checklist for verifying the skills of laypeople in CPR, without knowing which group the assessed deaf student had been allocated to.

A checklist was constructed and validated with 11 items referring to the steps to be performed in CPR, namely: position of the rescuer close to the victim’s shoulder, overlapping hands for CPR, hypothenar region over the center of the victim’s chest, rescuer’s shoulders at 90º from the victim’s chest, performing chest compressions, arms straight/without flexion during CPR, trunk movement to apply force in CPR, minimum depth of 5cm in compression, velocity 100 to 120 compressions per minute, with chest recoil allowed between compressions and no interruption of compressions. Each item contained the options “correct”, “incorrect” and “not performed” to be marked, for each item, by the evaluators who used it^(18)^.

### Data Analysis and Treatment

Data were double-typed in Microsoft Excel, and statistical tests were performed using the R software, version 3.1.1. Non-compliance with the variables’ normal distribution was confirmed using the Kolmogorov-Smirnov test. Descriptive statistics were used based on median values and the 25^th^ and 75^th^ percentiles, for numeric variables, and absolute frequencies and percentages, for nominal variables. The intergroup comparison was based on Mann-Whitney, Pearson’s chi-square for independence and Fisher’s exact tests. A significance level of 5% and a confidence interval of 95% were adopted for all tests.

### Ethical Aspects

The study complied with Resolution 466/12 of the Brazilian National Health Council. It was approved by the Research Ethics Committee of the *Universidade Federal do Ceará* in 2017, under Opinion 2,108,475. It should be noted that, for participants under the age of 18, participation was conditioned to signing the ICF by their guardian. In accordance with Resolution 466, the desire for participation by minors was also documented by signing the Informed Assent Form.

## RESULTS

A total of 113 participants participated in the study, of which 57 (50.5%) in the CG and 56 (49.5%) in the IG. IG and CG were homogeneous in terms of sociodemographic and clinical variables. In both groups, there was a predominance of single marital status (CG = 86%; IG = 89.3%), not having children (CG = 89.5%; IG = 91.1%), being in high school (CG = 86%; IG = 85.7%), not using a hearing aid (CG = 91.2%; IG = 91.1%) and having profound bilateral hearing loss (CG = 52.6%; IG = 51.8%), according to Table 1.

**Table 1.** Characterization and homogeneity of participants in the intervention and control groups. Fortaleza, CE, Brazil, 2018.

Variable		Control group	Intervention group	p*
	Response	n (%)	n (%)	
Sex	Male	30 (52.6)	26 (46.4)	0.518
	Female	27 (47.4)	30 (53.6)	
Marital status	Married	8 (14.0)	6 (10.7)	0.599
	Single	49 (86.0)	50 (89.3)	
Children	Yes	6 (10.5)	5 (8.9)	0.772
	No	51 (89.5)	51 (91.1)	
Education	Elementary school	8 (14.0)	8 (14.3)	0.977
	High school	49 (86.0)	48 (85.7)	
Use of hearing aid	Yes	5 (8.8)	5 (8.9)	0.977*
	No	52 (91.2)	51 (91.1)	
Hearing loss	Deep bilateral	30 (52.6)	29 (51.8)	0.947***
	Severe bilateral	11 (19.3)	13 (23.2)	
	Bilateral anacusis	1 (1.7)	0 (0.0)	
	Severe in one ear, deep in the other	7 (12.3)	5 (8.9)	
	Severe in one ear, moderate in the other	0 (0.0)	1 (1.8)	
	Deep in one ear, anacusis in the other	3 (5.3)	2 (3.6)	
	No conclusion on medical report	5 (8.8)	6 (10.7)	

*Chi-square for proportion; **Mann-Whitney; ***Fisher’s exact test.

Regarding the theoretical total correct answers, the groups were similar in the pre-test and showed a statistically significant difference in the immediate post-test and after 15 days (greater correct answers in IG). Regarding the total correct answers of practice, the groups differed in the pre-test (CG with better performance) were similar in the immediate post-test and showed differences again after 15 days, with higher values obtained, this time, in IG, as shown in Table 2.

**Table 2.** Theoretical and practical successes of the control and intervention groups in the pre-test, immediate post-test and post-test after 15 days. Fortaleza, CE, Brazil, 2018.

Variable	Control group	Intervention group	p*
	Median (75^th^ percentile)	Median (25^th^ and 75^th^ percentiles)	
Theoretical hits: knowledge			
Pre-test	5 (3–6)	4 (3–5)	0.635
Immediate post-test	7 (6–8)	8 (7–9)	0.035
Post-test after 15 days	7 (6–8)	8 (7–9)	0.026
Practical hits: skill			
Pre-test	7 (4–8.5)	6.25 (1–8)	0.031
Immediate post-test	10.5 (9.5–11)	10.5 (9.75–11)	0.77
Post-test after 15 days	10 (9.5–10.5)	10.5 (10–11)	0.014

*Mann-Whitney test.

The comparison of assessed items related to knowledge between CG and IG in the pre-test and post-test after 15 days, showed statistical significance in the items on victim positioning (p = 0.002) and rotation (p < 0.001), as shown in Table 3.

**Table 3.** Comparison of knowledge between the control and intervention groups in the pre-test and post-test after 15 days. Fortaleza, CE, Brazil, 2018.

Variable	Pre-test		p*	Post-test after 15 days		p
	Control group	Intervention group		Control group	Intervention group	
	n (%)	n (%)		n (%)	n (%)	
1- Identification	34 (59.6)	24 (42.9)	0.748	40 (70.2)	45 (80.4)	0.216*
2- Gravity	20 (35.1)	18 (32.1)	0.748	27 (47.4)	35 (62.5)	0.102*
3- Call for help	8 (14.0)	2 (3.6)	0.054	13 (22.8)	13 (23.2)	0.954*
4- SAMU number***	25 (43.9)	34 (60.7)	0.731	52 (91.2)	54 (96.4)	0.433**
5- Moment for compression	9 (15.8)	4 (7.1)	0.154	10 (17.5)	4 (7.1)	0.092*
6- Victim positioning	18 (31.6)	31 (55.4)	0.013	42 (73.7)	53 (94.6)	0.002*
7- Site for compression	41 (71.9)	35 (62.5)	0.282	56 (98.2)	56 (100.0)	0.311*
8- Rescuer’s position	23 (40.4)	29 (51.8)	0.224	51 (89.5)	47 (83.9)	0.387*
9- Compression strength	30 (52.6)	30 (53.6)	0.922	42 (73.7)	40 (71.4)	0.785*
10- Rotation	15 (26.3)	14 (25.0)	0.874	36 (63.2)	52 (92.9)	<0.001*
11- When to stop compression	26 (45.6)	22 (39.3)	0.499	35 (61.4)	34 (60.7)	0.942*

*Chi-square; **Fisher’s exact test. ***Emergency Mobile Care Service.

The comparison of items related to skill, between CG and IG, at the pre-test and post-test moments after 15 days, showed statistical significance in the item speed from 100 to 120 (p = 0.006), as shown in Table 4.

**Table 4.** Comparison of the skills between the control and intervention groups in the pre-test and post-test after 15 days. Fortaleza, CE, Brazil, 2018.

Variable	Pre-test		p*	Post-test after 15 days		p
	Control group	Intervention group		Control group	Intervention group	
	n (%)	n (%)		n (%)	n (%)	
1- Rescuer’s position	49 (86.0)	51 (91.1)	0.657**	57 (100.0)	56 (100.0)	§
2- Hand position	36 (63.2)	27 (48.2)	0.532**	55 (96.5)	56 (100.0)	0.496**
3- Hypothenar region in the thorax	28 (49.1)	27 (48.2)	1.02**	56 (98.2)	56 (100.0)	1.02**
4- Rescuer at 90° to the victim	44 (77.2)	36 (64.3)	0.132*	57 (100.0)	56 (100.0)	§
5- Start of compressions	46 (80.7)	38 (67.9)	0.112*	57 (100.0)	56 (100.0)	§
6- Straight arms	19 (33.3)	6 (10.7)	0.014**	43 (75.4)	46 (82.1)	0.743**
7- Trunk movement	26 (45.6)	11 (19.6)	0.014**	55 (96.5)	49 (87.5)	0.102**
8- Depth of 5 cm	33 (57.9)	28 (50.0)	0.38**	53 (93.0)	54 (96.4)	0.673**
9- Speed from 100 to 120	0 (0.0)	1 (1.8)	0.649**	9 (15.8)	24 (42.9)	0.006*
10- Return of the thorax	34 (59.6)	22 (39.3)	0.014**	53 (93.0)	55 (98.2)	0.496**
11- Uninterrupted compressions	26 (45.6)	19 (33.9)	0.138**	53 (93.0)	53 (94.6)	1.02**

*Chi-square; **Fisher’s exact test; §Impossibility of carrying out the test due to the similarity between groups, which made the analysis tend to zero.

## DISCUSSION

The results of this study showed that in both groups there was a predominance of single marital status, not having children and being in high school. In turn, a study carried out by Brazilian researchers using video to teach laypeople about CPR differed in that it presented a predominance of females (74.2%), with partners (59.1%) and corroborated education of more than eight years (56.1%)^(9)^.

The gap in knowledge and practice of death people observed in pre-tests reflects existing lack of preparation of this public in relation to the CPR theme. This reality is found among the lay public, since a study carried out with teachers and students in Nigeria pointed to a lack of knowledge in the school environment, as well as a study carried out with university students in China, whose results showed a gap in knowledge and practice in higher education students^(19,20)^. These findings point to the need for educational interventions with lay people to empower them on the subject. Among lay people, there is a need for studies including death people, since no studies were identified aimed at this target audience.

In the present study, the increase in correct answers from the pre- to the theoretical post-test was significant in most questions. However, in both groups, there was no significance in the question about the moment to perform CPR and, in the CG, in the question regarding the moment to stop the compressions. In the post-test after 15 days, the lowest rate of correct answers for both groups remained in the question about the moment to start compressions. This finding differs from a study carried out in Brazil, in which, after training for lay university students, participants improved with statistical significance (p < 0.01)^(21)^. Thus, it is confirmed that health education, mainly aimed at death people, must include not only the correct way of acting, but the correct moment so that early help is consistent with increased survival and reduced sequel in victims.

In the theoretical pre-test, the lowest rate of correct answers for both groups was in the question regarding the moment to call for help. A study carried out in Germany evaluated the response time for care and the outcome of victims of out-of- hospital CRA and concluded that a shorter interval for activation was associated with greater survival^(22)^. When considering that such conduct culminates in a reduction in mortality, it is important that this component of care for CRA victims be included in educational interventions and that its relevance be highlighted, especially in death people community.

The high theoretical and practical successes in the test after 15 days, of both groups, regarding the rescuer’s position to perform CPR, corroborate with a prospective observational study that assessed the teaching of laypeople about CPR in the United States, whose results showed that 63% of the people who participated in an educational intervention at a kiosk installed in an airport were able to learn and perform compressions in the correct position^(23)^. In view of these facts, it is pointed out that deaf people had an improvement in performance similar to that of lay people without hearing impairment present at the airport, which converges with the total capacity of deaf people to learn and, if necessary, provide quality help to CPR victims.

Two important variables in the practice of compressions are velocity and depth. The worst performances practiced by the deaf were related to CPR speed, both in the pre-test and in the test after 15 days. Similar results were found in a study with university students without hearing loss in Germany, in which the mean compression frequency was not significantly different between CG and IG (p = 0.17)^(24)^.

CPR training with lay people, reported in the literature, usually document short interventions^(23,25,26)^, and carrying out strength application in CPR, at the correct speed, is challenging due to the need for concentration and synchrony in body movement. Thus, correct speed requires repeating practice, which may not be possible in studies that obtain low accuracy values from laypeople. In the same way that laypeople achieved performance similar to professionals in the other items, they would probably also do it in speed, if there was an opportunity for repetition to improve practice.

As for depth, the correct answers almost doubled, in both groups so that it reached proportions of correct answers superior to 90% in the test after 15 days. Such improvement is also pointed out in the results of a study that assessed the use of an educational game about CPR in schools in Italy^(25)^. These results are relevant, since they make it possible to infer that any previous fear of causing injury by applying force was overcome in the post-test.

When considering that knowledge and skill deteriorate over time, it is expected that the number of correct answers will show a decline when measured more than once in post-tests^(26)^. However, in the present study, the positioning the victim in a rigid and flat place, positioning the hypothenar region in the center of the chest, keeping arms straight and shoulders at 90° with the victim’s chest and trunk movement to apply force in CPR variables had increasing hits.

The non-reduction of correct answers over time, observed among deaf people, referring to visual content and information, can be explained by sensorineural behavior during information processing and brain plasticity. According to a study carried out in London, hearing people process auditory information from the superior temporal cortex, while in deaf people, this brain area starts to participate in cognitive tasks and is activated in the face of visual stimuli^(27)^.

In some aspects, the video proved to be as effective as the lecture with a practical demonstration of CPR, but in others, the effectiveness of the video was superior. When analyzing each assessed item, it was observed, regarding knowledge, similarity between the groups in nine of the eleven questions, with difference only in relation to the questions about the rescuer relay and the positioning of the victim in a rigid and flat place, in which IG was superior.

Regarding practice, in the pre-test, the groups differed in only three questions (on straight arms and rescuer trunk movement), all with more correct answers in CG. After 15 days, the improvement in IG led the groups to be similar in all items, except for speed, in which IG had more correct answers. Such findings point to the successful use of video as a didactic resource, which can be used in self-instruction, in the mass multiplication of information and in content provision even in the absence of a health professional.

Thus, the present study has an unprecedented character, a pioneer in the national and international context so that its findings contribute to the practice of professionals involved in health education regarding CRA. Among such professionals, nurses stand out, since they have health education inherent to their professional activities. They work at all levels of health care in which deaf people are assisted, integrating the multidisciplinary team of pre-hospital and in-hospital emergency services, for which the multiplication of information for lay people about CPR impacts on the indicators of survival.

The limitation of this study regarding external validity concerns its performance in a region of the country and with deaf people who are inserted in the school environment, so that other results can be obtained in methodological replication in other regions or with deaf people who are not students. Another limitation regarding the external validity is related to a 15-day interval between the pre- and post-test so that the results found may differ from those found in future studies that use other intervals between pre- and post-tests. As for internal validity, the limitation referred to the impossibility of blinding the researcher and participants.

The present study has relevant results for health professionals involved in teaching CPR, who may consider using the video for health education on the subject for deaf people. Among such professionals, nursing stands out as, inserted in emergency services and active in health education, it can use video in teaching, research and extension activities. It is pertinent that other studies consider the varied use of video: in other scenarios, in interventions with more opportunity for participants to practice, with longer time interval monitoring and that compare its use with hearing and deaf people.

## CONCLUSIONS

The video proved to be effective in increasing deaf people’s knowledge and skills about CPR. It was found that both the lecture with practical demonstration of CPR and the educational video were effective for theoretical and practical training in CPR, but the educational video was associated with greater retention of knowledge after 15 days.
